# Photodetectors Based on Micro-nano Structure Material

**DOI:** 10.3389/fchem.2021.832028

**Published:** 2022-01-12

**Authors:** Yu Yu, Wuyue Wang, Weihua Li, Gong Wang, Yulei Wang, Zhiwei Lu, Sensen Li, Wanli Zhao, Yuhai Li, Tongyu Liu, Xiusheng Yan

**Affiliations:** ^1^ Center for Advanced Laser Technology, Hebei University of Technology, Tianjin, China; ^2^ Hebei Key Laboratory of Advanced Laser Technology and Equipment, Tianjin, China; ^3^ Weihai Photonics Information Technology Lab Co., Ltd., Shandong, China; ^4^ Science and Technology on Electro-Optical Information Security Control Laboratory, Tianjin, China

**Keywords:** photodetector, micro-nano structure, performance, fabrication, applications

## Abstract

Photodetectors converting optical signals into electrical signals have been widely utilized and have received more and more attention in scientific research and industrial fields including optical interconnection, optical communication, and environmental monitoring. Herein, we summarize the latest development of photodetectors with different micro-nano structures and different materials and the performance indicators of photodetectors. Several photodetectors, such as flexible, ultraviolet two-dimensional (2D) microscale, and dual-band photodetectors, are listed in this minireview. Meanwhile, the current bottleneck and future development prospects of the photodetector are discussed.

## Introduction

Traditionally, many ordered micro-nano structures have been emerged in natural organisms. Particularly, for plants, the micro-nano structures can guide water droplets to roll freely on their leaves or stay on petals (Zhang et al., 2012a; Zhang et al., 2012b). Inspired by the micro-nano structure of organisms in nature, a growing number of researchers have focused on the application of micro-nano structures in scientific research. Micro-nano structure arrays are widely used in PDs due to their unique order-related characteristics of ordering or patterning. Photodetectors (PDs) that convert optical signals into electrical signals have been widely used and paid more and more attention in scientific research and industrial fields such as biological detection, optical communications, and environmental monitoring (Teng et al., 2018). At present, the main materials used in photodetectors for detecting ultraviolet (UV) to near-infrared spectra are crystalline-Si and III–V (Deng et al., 2015). The reason why micro-nano structure arrays can be rapidly developed in the fields of science and industry is because they can improve the efficiency of light scattering, reduce light reflection efficiency, and extract light better and surface-to-volume ratio of organic light-emitting devices (OLED) (Gao et al., 2021), resulting in the photon supersurface (Li et al., 2021). Different performances can be achieved by changing the size, distribution and different result shapes of each micro/nano structure.

At present, the production of photodetectors with different properties mainly depends on the development of nanotechnology, including template method, photolithography, self-assembly and other methods. Many new types of photodetectors have been fabricated through the above technology, such as high-sensitivity phototransistors (Hai et al., 2015), flexible photodetectors, self-powered ultraviolet detectors with heterojunction nanowire arrays, pyramid array photodetectors, and dual-band detection array van der Waals. Broadband detector, sensitive infrared photodetector (IRPD). Flexible photodetectors (PD) have received more and more attention due to their structural characteristics in many applications, such as wearable optoelectronic devices, bendable imaging sensors and implanted optoelectronic devices (Deng et al., 2019). The self-powered photodetector array based on organic-inorganic heterojunction has unique flexibility and stability due to its special structure, which makes it widely used in optical imaging (Ouyang et al., 2017). Among them, sapphire is used as the substrate, and the pyramidal structure of MoS_2_ as the material can enhance the strong interaction, thereby improving the performance of the optoelectronic device (Wang P et al., 2017). Dual-band photodetector for dual-color imaging. By introducing a strong local field, the dark current is reduced, so that the photo-generated carrier separation efficiency is increased, so that the dual-band detection capability of the broadband photodetector is improved (Wang S et al., 2017). Each structure has its own unique properties, and photodetectors of different structure types are used in different optical fields. Therefore, it is particularly important to study the characteristics of different structures in photodetectors.

In this minireview, we focus on the latest developments in the application of different structures to photodetectors. Summarized typical cases, such as flexible photodetectors, ultraviolet photodetectors, high-sensitivity ultra-fast response array photodetectors, high-performance dual-band photodetectors, are discussed the challenges faced by photodetectors and future prospects.

## Flexible Photodetectors Based on Micro-nano Structure Material

**FIGURE 1:**
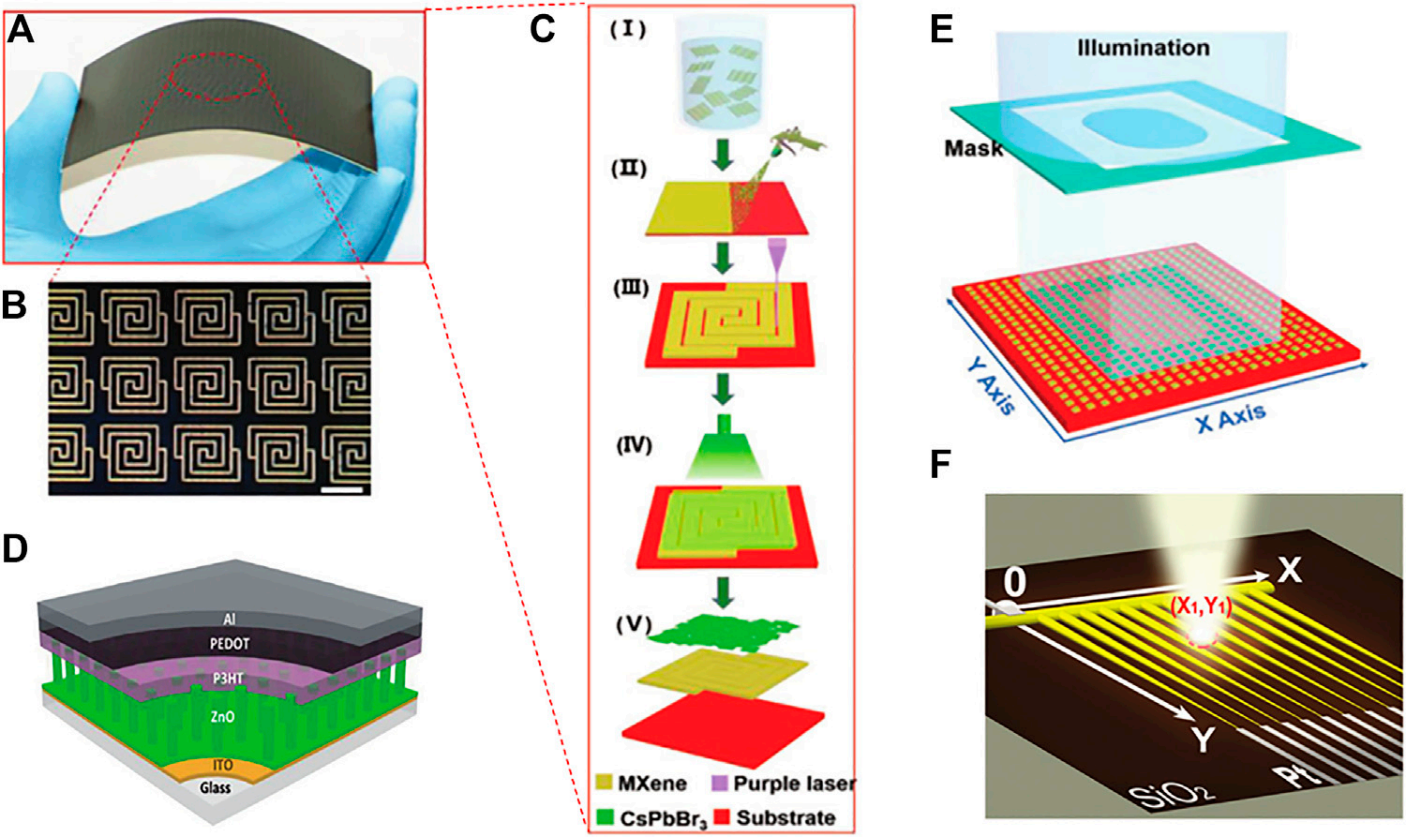
**(A)** Exhibition of large-area arrays MXene electrode. (B) Micro pattern corresponding to **(A)**. **(C)** Schematic diagram of flexible photodetector processing process. **(D)** Schematic of the P3HT/ZnO heterojunction photodetector. **(E)** Schematic diagram of a digital sensor based on a large-area array photodetector. **(F)** Schematic diagram of the CdS branched photodetector.

Flexible photodetectors (FPDs) have received more and more attention due to their structural characteristics in many applications, such as Optical communication, industrial automatic control, medical sensor monitoring and intelligent robots and implanted optoelectronic devices. For large-scale manufacturing of FPD processing technology and its use of complex high-vacuum technology, which makes the functional function of its active materials need to achieve a good match. Traditional PDs are usually fabricated on rigid substrates, which seriously hinders their rapid development in the fields of flexible optoelectronic devices such as industrial automatic control, medical sensor monitoring, and implantable optoelectronic devices (Liu et al., 2017). In contrast to rigid PDs, FPDs can meet the needs of industrial automatic control, medical sensor monitoring, and implantable optoelectronic devices. However, it has high optoelectronic performance and good flexibility. It is integrated in an optoelectronic device, which is still a huge challenge for technical processing so far (Hossain et al., 2018). In 2019, Deng et al. demonstrated all spray processable and large area FPDs on plain paper based on two-dimensional (2D) CsPbBr_3_ nanosheets and conductive Ti_3_C_2_T_x_ (MXene) (Deng et al., 2019). Figure 1A is its large-area MXene electrode array, and Figure 1B is its microscopic model. The production process is shown in Figure 1C. Due to the Figure 1C good conductivity of MXene, the higher crystallinity of 2D CsPbBr_3_, and their good matching with the work function, on/off current ratio of the PD reaches 2.3×10^3^, and the light response reaches 18 ms. At the same time, Jones’ detection rate (D*) reached 6.4 × 10^8^ under 10 V bias and the response rate (R) reached 44.9 mA W^−1^. In addition, the PD still has good flexibility and stability after being bent 1,500 times.

## UV Micro-nano Structure Material Photodetectors

As an electronic sensor that converts electrical signals into light signals, ultraviolet (UV) photodetectors have good application prospects in many ways such as medicine, biology, flame monitoring, optical communication, day/night monitoring, and missile detection (Koppens et al., 2014; Chen et al., 2016; Liang et al., 2016; Esopi et al., 2017; Zhang et al., 2017). In 2017, Bangsen et al. have constructed a self-powered UV photodetector based on p-P3HT/n-ZnO nanowire array heterojunction (Ouyang et al., 2017). The schematic diagram of the photodetector is shown in Figure 1D. The light response of this PDs at *λ* = 365 nm can reach 125 μA W^−1^ to 0.84 mW cm^−2^, and its response and recovery time are both less than 100 ms. Moreover, a photodetector array with 16 pixels is successfully demonstrated for light imaging of complex patterns, such as number-shape and cross-shape. This study provides a practical solution to achieve large-scale UV imaging by integrating inorganic–organic hybrid photodetector into self-powred array configuration.

## 2D Microscale Position-Sensitive Photodetectors

With the continuous improvement of integration and the reduction of the size of nano-devices, two-dimensional micro-scale position sensitive detectors (PSD) have received more and more attention. Nowadays, two-dimensional materials have gradually become mainstream due to their excellent optoelectronic properties (An et al., 2013; Gan et al., 2013; Lopez-Sanchez et al., 2013; Wang et al., 2013; Baugher et al., 2014; Furchi et al., 2014a; Furchi et al., 2014b; Gong et al., 2014; Lee et al., 2014; Ross et al., 2014; Zhang et al., 2014; Li et al., 2015; Wang et al., 2015a; Wang et al., 2015b; Youngblood et al., 2015; Yue et al., 2015; Long et al., 2016). Figure 1E is a schematic diagram of a digital sensor based on a large area array PD. In 2018. For the first time, Chen et al. have grown a well-defined hollow spherical nanoshell array of two-dimensional transition metal aluminum dichloride (TMDC) nanomaterials for MoSe_2_ and MoS_2_ through chemical vapor deposition technology (Chen et al., 2018). The responsivity of the MoSe_2_ hollow sphere photodetector reaches 8.9 A W^−1^, which is about 10 times that of the MoSe_2_ dense film (0.9 A W^−1^). At the same time, the hollow sphere PDs has a fast response and recovery speed and *λ* = 365 nm irradiation good durability at wavelengths. Xu et al. designed a high-sensitivity ultra-fast response array photodetector based on a new two-dimensional lead iodide perovskite crystal (Xu et al., 2019). The array photodetector achieves high phodetectivities (6.3 × 10^12^ Jones) responsivities (≈47 A W^−1^) and low dark current (≈2.4 × 10^–11^ A). In 2019, Li et al. developed a method capable of heterogeneous integration of atomically thin 2D crystal arrays for system-on-chip electrons on a planar patterned silicon substrate (Li et al., 2019). In addition, multi-channel devices with good optical and electrical characteristics are widely used in system-on-chip (Hao et al., 2019). Schematic diagram of CdS branch photodetector. As shown in Figure 1F. Hao et al. based on a highly ordered comb-shaped CdS nanowire array with tapered branches, a one-step synthesis strategy is used to achieve high-resolution 2D position-sensitive photodetection through the variable resistance of multiple lines and the variable optical response of different parts Device. The tapered branch can accurately identify the position of the incident light in each area of the nanowire array according to the change of the photocurrent. In addition to the above-mentioned traditional photodetectors, more and more complex photodetectors are gradually being used in many fields. Multicolor photodetectors have a wide range of applications in the fields of imaging, (Schermelleh et al., 2008; Sang et al., 2013), medical treatment, (Keller et al., 2001), astronomical observations (Fontana et al., 2004) and military applications (Tribolet and Destefanis, 2005). Ji et al. used interface engineering technology to develop an ultraviolet-visible multicolor photodetector based on n-Si (111)/TiO_2_ nanorod array heterojunction (Tao et al., 2016). The photodetector is manufactured through continuous processes such as chemical etching, magnetron sputtering, hydrothermal growth and assembly. In the case of low reverse bias voltage (0∼-2 V), only photo-generated electrons in TiO_2_ can pass through the low ΔEC barrier, and the device only responds to ultraviolet light.

## Dual-Band Micro-nano Structure Material Photodetectors

In the past few decades, in the field of dual-color detection technology (DCDT), people have made significant progress by introducing new materials such as quantum dots and superlattices (Martyniuk et al., 2014; Kufer et al., 2015; Lei et al., 2015; Hoang et al., 2016). Wang et al. designed a dual-band photodetector with high-performance dual-color imaging and wafer-level 2D GaSe/GaSb van der Waals vertical heterostructure based on molecular beam epitaxial growth (Wang P et al., 2017). By introducing a strong local field, the dark current is reduced, so that the photo-generated carrier separation efficiency is increased, so that the dual-band detection capability of the broadband photodetector is improved. Ultrasensitive visible and infrared specific detectivities reach up to 2.2 × 10^12^ and 1.3 × 10^12^ Jones, respectively, and an excellent external quantum efficiency up to 50% is obtained with microsecond response speed, which is expected due to its photovoltaic mechanism for free-carrier generation. This new type of heterogeneous photodiode also has the good photoresponsibility of the two-dimensional material GaSe in the visible light band and also have the excellent photodetection performance of the traditional GaSb in the infrared light band. It provides a new way for two-dimensional materials to be used in actual room temperature applications.

## Conclusion and Outlook

In this minireview, we summarize the performance indicators of photodetectors with different structures. Such as flexible photodetectors, ultraviolet photodetectors, high-sensitivity ultra-fast response array photodetectors, and high-performance dual-band photodetectors are listed. Although these devices have reached very good performance indicators. However, there are still many challenges to realize low-cost and large-scale preparation. However, with the rapid development of micro-nano manufacturing technology, photodetectors with good performance will be better used in electronic information, optical communications, environmental monitoring and other fields.
